# Real-Life Indications of Interleukin-1 Blocking Agents in Hereditary Recurrent Fevers: Data From the JIRcohort and a Literature Review

**DOI:** 10.3389/fimmu.2021.744780

**Published:** 2021-11-11

**Authors:** Caroline Vinit, Sophie Georgin-Lavialle, Aikaterini Theodoropoulou, Catherine Barbier, Alexandre Belot, Manel Mejbri, Pascal Pillet, Jana Pachlopnik, Sylvaine Poignant, Charlotte Rebelle, Andreas Woerner, Isabelle Koné-Paut, Véronique Hentgen

**Affiliations:** ^1^ General Pediatrics, Versailles Hospital, Versailles, France; ^2^ CEREMAIA (French reference center for auto-inflammatory diseases and inflammatory amyloidosis), Kremlin-Bicêtre, France; ^3^ Department of Internal Medicine, Sorbonne University, Tenon Hospital (APHP), Paris, France; ^4^ Pediatric Immuno-Rheumatology of Western Switzerland, Department Women-Mother-Child, Lausanne University Hospital, Lausanne, Switzerland; ^5^ Pediatric Immuno-Rheumatology Department, University Hospital, Geneva, Switzerland; ^6^ Pediatric Immunology, Albert Michallon Hospital, Grenoble, France; ^7^ Pediatric Nephrology Rheumatology and Dermatology, CHU Lyon, Lyon, France; ^8^ RAISE (Centre de référence des rhumatismes inflammatoires et maladies auto-immunes systémiques de l’enfant), Paris, France; ^9^ Pediatrics and Immunology, CHU Pellegrin, Bordeaux, France; ^10^ Pediatric Immunology, Kinderspital, Zurich, Switzerland; ^11^ General Pediatrics, CHU Nantes, Nantes, France; ^12^ Pediatric Cardiology and Rheumatology, UKBB Hospital, Bâle, Switzerland; ^13^ Pediatric Rheumatology Department, Bicêtre Hospital, APHP, University of Paris Saclay, Kremlin Bicêtre, France

**Keywords:** autoinflammatory diseases, indications, treatment, hereditary recurrent fevers, interleukin-1 blockers, anakinra, canakinumab

## Abstract

**Background:**

Interleukin (IL)-1 inhibitors represent the main treatment in patients with colchicine-resistant/intolerant familial Mediterranean fever (crFMF), mevalonate kinase deficiency (MKD), and tumor necrosis factor receptor-associated periodic syndrome (TRAPS). However, the reasons for the use of IL-1 inhibitors in these diseases are still not completely clarified.

**Objective:**

Identify real-life situations that led to initiating anakinra or canakinumab treatment in hereditary recurrent fevers (HRFs), combining data from an international registry and an up-to-date literature review.

**Patients and Methods:**

Data were extracted from the JIRcohort, in which clinical information (demographic data, treatment, disease activity, and quality of life) on patients with FMF, MKD, and TRAPS was retrospectively collected. A literature search was conducted using Medline, EMBASE, and Cochrane databases.

**Results:**

Complete data of 93 patients with HRF (53.8% FMF, 31.2% MKD, and 15.1% TRAPS) were analyzed. Data from both the registry and the literature review confirmed that the main reasons for use of IL-1 blockers were the following: failure of previous treatment (n = 57, 61.3% and n = 964, 75.3%, respectively), persistence of disease activity with frequent attacks (n = 44, 47.3% and n = 1,023, 79.9%) and/or uncontrolled inflammatory syndrome (n = 46, 49.5% and n = 398, 31.1%), severe disease complication or associated comorbidities (n = 38, 40.9% and n = 390, 30.4%), and worsening of patients’ quality of life (n = 36, 38.7% and n = 100, 7,8%). No reasons were specified for 12 (16.4%) JIRcohort patients and 154 (12%) patients in the literature.

**Conclusion:**

In the absence of standardized indications for IL-1 inhibitors in crFMF, MKD, and TRAPS, these results could serve as a basis for developing a treat-to-target strategy that would help clinicians codify the therapeutic escalation with IL-1 inhibitors.

## Introduction

Hereditary recurrent fevers (HRFs) belong to the large group of autoinflammatory diseases (AIDs), whose name was first proposed by McDermott et al. in 1999 (1). This term describes inflammatory febrile attacks, with minimal involvement of the adaptive immune system in opposition to so-called autoimmune diseases.

Advances in the pathophysiological knowledge on HRFs have led to targeted therapies to improve patients’ quality of life, reduce the inflammatory response, and prevent amyloidosis. Based on the excess production of interleukin (IL)-1, the first patients with cryopyrin-associated periodic syndrome (CAPS) effectively treated with the IL-1 receptor antagonist anakinra were described in 2003 (2). The benefit of IL-1-blocking agents has subsequently been reported in the four prototypic monogenic AIDs (3–7). Anakinra received the market authorization (MA) for CAPS in 2013 and colchicine-resistant/intolerant familial Mediterranean fever (crFMF) in April 2020 in Europe (8). In addition, several studies reported its efficacy also in mevalonate kinase deficiency (MKD) and tumor necrosis factor (TNF) receptor-associated periodic syndrome (TRAPS) patients (9–11). Canakinumab is licensed in adults and children from 2 years of age in the European Union (i) for first-line maintenance treatment in CAPS since 2009 and (ii) for familial Mediterranean fever (FMF), MKD, and TRAPS since 2017 (12). However, regulatory authorities’ indications may vary across countries.

IL-1 inhibitors are indicated for patients with (i) severe clinical manifestations, (ii) failure or severe side effects of treatment in MKD and TRAPS attacks, or (iii) crFMF (13). However, the precise clinical and biological criteria leading to the use of IL-1 inhibitors in these diseases are still unclear.

The main objective of our study was to identify actual criteria for the use of IL-1-blocking agents (anakinra and canakinumab) in patients with crFMF, MKD, or TRAPS using data from the Juvenile Inflammatory Rheumatism (JIR)cohort and an updated literature review.

## Patients and Methods

### JIRcohort

The JIRcohort is an observational and multicenter international registry created in 2013 to collect data on patients with juvenile inflammatory rheumatic diseases. The cohort operates in specific modules; the module on AID has been running since April 2016. The participating centers can record both retrospective and prospective data, especially during follow-up visits. Patients are included in the JIRcohort after information and verification that they (or their legal guardian) are not opposed to the study and storage of their data. The JIRcohort protocol was approved by the French Ethics Committee (CCTIRS) on April 21, 2015 (decision number 14.302). The electronic form of the JIRcohort was approved by the National Commission of data processing and liberties (CNIL) on March 27, 2015 (decision number DR-2015-218).

Our survey collected only data from patients with FMF, MKD, and TRAPS treated with IL-1 inhibitors (anakinra and/or canakinumab). Selected patients came from 12 different centers in France and Switzerland. Since the HRFs Genoa criteria (14) were not available for all patients, the diagnosis of FMF, MKD, or TRAPS was established by the examining physician and investigator. Demographic data (age at diagnosis, genetics, comorbidities), therapeutic data, and data assessing disease activity and quality of life were collected at the last visit before starting IL-1 therapy. The presence of likely pathogens or pathogenic variants defined a confirmatory genotype according to the International Study Group for Systemic Autoinflammatory Diseases classification rules described elsewhere (15). Data evaluating disease activity included (i) biological features [C-reactive protein (CRP) and serum amyloid A protein (SAA) levels], (ii) flares’ characteristics (duration and frequency defined as more than three in 6 months), and (iii) the visual analog scale (VAS). The patient and physician rated this tool from 0 to 10, and an active disease was defined by a median VAS ≥ 3/10. The Auto-Inflammatory Diseases Activity Index (AIDAI) score validated and used in clinical practice was not, at this time, recorded in the JIRcohort. Data evaluating the quality of life included (i) number of hospitalizations or consultations, (ii) school or work impact (number of missing days, stress of exams triggering an attack, reduction of working times), (iii) severe daily asthenia and ongoing symptoms, and (iv) psychosocial impact (disease-related anxiety/depression). Therapeutic data included previous treatment(s), efficiency, and toxicity. Anakinra was prescribed continuously with daily injections or as on-demand treatment at the time of an attack. Patients without a follow-up visit recorded in the JIRcohort were excluded. The data extraction took place on May 15, 2020.

### Literature Review

A literature search on the indications for IL-1 inhibitors in HRFs was performed using Medline, EMBASE, and Cochrane databases with mesh terms (Ilaris OR Canakinumab)/(Kineret OR Anakinra)/(IL-1 inhibitors) AND (familial Mediterranean fever OR FMF)/(mevalonate kinase deficiency OR MKD OR HIDS)/(periodic tumor necrosis factor receptor-related syndrome OR TRAPS). Additional research was conducted within the references of the retrieved papers. We identified 359 publications through this research before May 2020. We assessed all titles, abstracts, and full-length articles identified. Publications were eligible if they contained either data on anakinra and/or canakinumab in patients with FMF, MKD, or TRAPS as defined by the Tel Hashomer criteria (16) or a genetic analysis or a mevalonic aciduria dosage. After removing duplications, we excluded articles (i) that were not published in English nor French, (ii) with unavailable abstract or full text, and (iii) without data on canakinumab nor anakinra. After this process, 112 articles (randomized controlled trials, non-randomized trials, cohort studies, case reports, and case series) remained for analysis and were included in the study. Possible indications for IL-1 inhibitors were identified with data evaluating comorbidities, complications, disease activity, previous treatment(s), and tolerance.

## Results

### Patients

#### JIRcohort

At extraction date, 613 HRF patients were included in the JIRcohort: 524 FMF patients, 45 MKD patients, and 44 TRAPS patients. Ninety-three patients, male-to-female ratio 40/53, were treated with IL-1 inhibitors and had a follow-up in French (N = 71, 76.3%) and Swiss (N = 22, 30.1%) centers. Fifty (53.8%) had a diagnosis of FMF, 29 (30.9%) of MKD, and 14 (14.9%) of TRAPS. First symptoms appeared at a median age of 3.5 (2–35) years for FMF, 0.4 (5–40) years for MKD, and 2 (1–17) years for TRAPS. The median delay to diagnosis was the longest for patients with TRAPS [14.5 (0.2–47) years]. The median time of follow-up was 9 (1–60) years for all patients. Sixteen (17.2%) patients had associated secondary amyloidosis, mainly adults with FMF.

For FMF, 31 patients (62%) had a confirmatory genotype, five patients (10%) had a non-confirmatory genotype, 10 patients (20%) were not tested, and the genotype was not specified in four patients (8%). For MKD, 18 patients (62.1%) had a confirmatory genotype, three patients (10.3%) had a non-confirmatory genotype, four patients (13.8%) were not tested, and the genotype was not specified in four patients (13.8%). For TRAPS, seven patients (50%) had a confirmatory genotype, four patients (28.6%) had a non-confirmatory genotype, two patients (14.3%) were not tested, and the genotype was not specified in one patient (7.1%). The main clinical characteristics are detailed in **Table 1**.

**Table 1:** Main characteristics: JIRcohort and literature review. IL, interleukin; FMF, familial Mediterranean fever; MKD, mevalonate kinase deficiency; TRAPS, tumor necrosis factor receptor-associated periodic syndrome.

Characteristics	FMF		MKD		TRAPS	
N patients (%)	JIRcohort N = 50	Literature N = 790	JIRcohort N = 29	Literature N = 265	JIRcohort N = 14	Literature N = 226
**AID, median (interval, year)**						
Age at diagnosis	9 (1.5–61)	18 (2–70)	6.5 (0.3–60)	11.5 (1–74)	21.5 (2–47)	18 (2–70)
Diagnostic time	2 (0–48)		5 (0–54)		14.5 (0.2–45)	
Follow-up time	15 (1–60)	17 (0.5–60)	7 (0.5–18)	9 (0.5–43)	6 (1–13)	10 (1–57)
N attack/year		24 (10–60)		15 (3–24)		10 (1–36)
**Genetic status**						
Confirmatory	31 (62)	593 (75.1)	18 (62.1)	250 (94.3)	7 (50)	146 (64.6)
Non-confirmatory	5 (10)	45 (5.7)	3 (10.3)	1 (0.4)	4 (28.6)	21 (9.3)
Not specified	4 (8)	152 (19.2)	4 (13.8)	14 (5.3)	1 (7.1)	59 (26.1)
Not tested	10 (20)	0	4 (13.8)	0	2 (14.3)	0
**Amyloidosis**	13 (26)	320 (40.5)	1 (3.4)	26 (9.8)	2 (14.3)	12 (5.3)
**Anti-IL-1 treatment**						
Anakinra	26 (52)	496 (62.8)	6 (20.7)	144 (54.3)	7 (50)	114 (50.4)
On demand	4 (7.8)	20		16		3
Canakinumab	14 (28)	214 (27.1)	11 (37.9)	93 (35.1)	3 (21.4)	108 (47.8)
Both	10 (20)	80 (10.1)	12 (41.4)	28 (10.6)	4 (28.6)	4 (1.8)
Ana > Can	10	77	12	28	2	4
Can > Ana	0	3	0	0	2	0

#### Literature Review

One thousand two hundred eighty-one patients were identified in 112 articles, including 75 case reports and case series (11, 11, 17–88), 31 cohort studies (10, 89–119), four phase II trials (99, 120–122), and two randomized placebo-controlled trials (9, 12). Seven hundred ninety patients (61.7%) were diagnosed with FMF, 265 (20.7%) with MKD, and 226 (17.6%) with TRAPS. Five hundred sixty-nine came from central Europe (44.4%), 520 from the Eastern Mediterranean area (Israel, Turkey, 40.6%), six from Asia (China, Japan, 0.5%), three from the United States and Australia (0.2%), and 185 from an international registry (Eurofever, 14.4%). Three hundred fifty-eight patients (27.9%) had secondary amyloidosis, mostly FMF (n = 320/790).

For FMF, 593 patients (75.1%) had a confirmatory genotype, 45 patients (5.7%) had a non-confirmatory genotype, and the genotype was not specified in 152 patients (19.2%). For MKD, 250 patients (94.3%) had a confirmatory genotype, one patient (0.4%) had a non-confirmatory genotype, and the genotype was not specified in 14 patients (5.3%). For TRAPS, 146 patients (64.6%) had a confirmatory genotype, 21 patients (9.3%) had a non-confirmatory genotype, and the genotype was not specified in 59 patients (26.1%). All patients were tested in the literature review for FMF, MKD, and TRAPS. The main characteristics are reported in **Table 1**.

#### Interleukin-1-Blocking Agents

Anakinra was the principal IL-1-blocking agent reported in the literature (n = 754, 58.9%), sometimes used as on-demand therapy (n = 39/754 patients in the literature and 39/93 in the JIRcohort). Patients who received both IL-1 inhibitors were almost exclusively treated with anakinra before canakinumab (n = 109/112 patients in the literature and 24/26 in the JIRcohort). In the JIRcohort, IL-1 inhibitors were mainly used off-label for 87.1% of patients (n = 81/93), including 95.8% in France (n = 68/71) and 59.1% in Switzerland (n = 13/22). Median age at the first use of an IL-1 inhibitor occurred later for FMF patients [30 (4–82) years] than for MKD [11.5 (0.7–57) years] or TRAPS [13 (3–51) years] patients. Reasons for use of IL-1 inhibitors could be classified into four main categories and were similar in both JIRcohort and literature, i.e., failure of previous treatment, serious complications or comorbidities, the persistence of clinical and/or biological activity, and decreased quality of life (**Tables 2**, **3** **)**.

**Table 2:** IL-1 blockers’ indications: JIRcohort (population N = 73). IL, interleukin; FMF, familial Mediterranean fever; MKD, mevalonate kinase deficiency; TRAPS, tumor necrosis factor receptor-associated periodic syndrome.

JIRcohort	FMF			MKD			TRAPS		
N patients (%)	Anakinra n = 26	Canakinumab n = 14	Both n = 10	Anakinra n = 6	Canakinumab n = 11	Both n = 12	Anakinra n = 7	Canakinumab n = 3	Both n = 4
**Preceding treatment with Colchicine**									
Failure	22 (84.6)	13 (92.9)	6 (60)						
Toxicity	1 (15.4)	3 (21.4)	2 (20)						
**Other preceding treatments**									
Failure	3 (11.5)	1 (7.1)	1 (20)	2 (33.3)	2 (18.2)	2 (16.7)	3 (42.9)	0	2 (50)
Toxicity	0	0	0	0	0	1 (8.3)	0	0	1 (25)
Refusal	0	0	0	0	1 (9.1)	0	0	0	0
None specified	0	1 (7.1)	0	2 (33.3)	1 (9.1)	2 (16.7)	1 (14.3)	2 (66.7)	0
**Serious clinical complication**	18 (69.2)	4 (28.6)	7 (70)	2 (33.3)	4 (36.4)	0	2 (28.6)	0	1 (25)
**Clinical activity**									
Frequent attacks	17 (65.4)	4 (28.6)	6 (60)	0	5 (45.5)	6 (50)	4 (57.1)	0	2 (50)
Clinical score	3 (11.5)	4 (28.6)	0	1 (16.7)	3 (27.3)	0	0	1 (33.3)	0
**Biological activity**									
Between attacks	17 (65.4)	4 (28.6)	6 (60)	3 (50)	6 (54.5)	3 (25)	3 (42.9)	1 (33.3)	3 (75)
**Decreased quality of life**	10 (38.5)	5 (35.7)	4 (40)	1 (16.7)	7 (63.6)	5 (41.7)	1 (14.3)	1 (33.3)	2 (50)
**No indication specified**	0	2 (14.3)	0	0	1 (9.1)	5 (41.7)	1 (14.3)	2 (66.7)	1 (25)

**Table 3:** IL-1 blockers’ indications: literature review (population N = 1,210). IL, interleukin; FMF, familial Mediterranean fever; MKD, mevalonate kinase deficiency; TRAPS, tumor necrosis factor receptor-associated periodic syndrome.

Literature	FMF			MKD			TRAPS		
N patients (%)	Anakinra n = 496	Canakinumab n = 214	Both n = 80	Anakinra n = 144	Canakinumab n = 93	Both n = 28	Anakinra n = 114	Canakinumab n = 108	Both n = 4
**Preceding treatment with Colchicine**									
Failure	449 (90.5)	205 (95.8)	77 (96.2)						
Toxicity	142 (28.6)	27 (12.6)	3 (3.8)						
**Other preceding treatments**									
Failure	63 (12.7)	25 (11.7)	16 (20)	92 (63.9)	11 (11.8)	14 (50)	52 (36.1)	27 (25)	2 (50)
Toxicity	0	0	0	1 (0.7)	0	0	7 (6.1)	0	2 (50)
Refusal	0	0	0	2 (1.4)	0	0	0	0	0
**Serious clinical complication**	229 (46.2)	34 (15.9)	42 (52.5)	51 (35.4)	4 (4.3)	7 (25)	19 (16.7)	2 (1.9)	2 (50)
**Clinical activity**									
Frequent attacks	429 (86.5)	205 (95.8)	65 (81.3)	100 (69.4)	85 (91.4)	18 (64.3)	45 (39.5)	73 (67.6)	3 (75)
Clinical score	108 (21.8%)	86 (40.2%)	13 (16.3%)	26 (18)	78 (83.9)	7 (25)	29 (20.1)	86 (82.4)	4 (10)
**Biological activity**									
During attacks	47 (9.5%)	103 (48.1%)	12 (15%)	52 (36.1)	78 (83.9)	17 (60.7)	6 (5.2)	47 (43.5)	3 (75)
Between attacks	165 (33.3%)	46 (21.5%)	26 (32.5%)	72 (0.5)	7 (7.5)	8 (28.6)	45 (39.5)	27 (25)	0
**Decreased quality of life**	44 (8.9%)	0	2 (2.5%)	35 (24.3)	7 (7.5)	4 (14.3)	8 (7)	0	0
**No indication specified**	32 (6.5%)	6 (2.8%)	0	27 (18.8)	0	0	55 (48.2)	34 (31.5)	0

### Previous Treatments

#### Colchicine and Familial Mediterranean Fever

##### JIRcohort

Forty-one FMF patients (82%) were identified as colchicine-resistant, even if this characteristic was not explained in detail in the JIRcohort. Twenty-seven patients (54%) had frequent attacks according to their treating physician, of whom eight had 0.25–1 attack/month, eight patients had 1–3 attacks/month, and three had ≥4 attacks/month. Colchicine was continued in combination with the IL-1 inhibitors in almost all patients (n = 46, 92%). Colchicine toxicity was reported in five (10%) patients (diarrhea, n = 2; polyneuropathy, n = 2; and rhabdomyolysis, n = 1).

##### Literature Review

**Figure 1:**
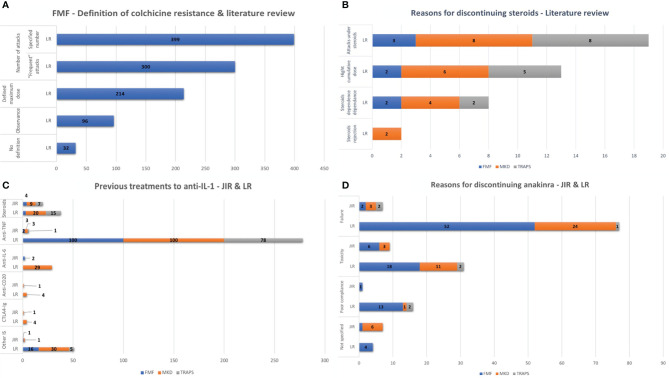
**(A)** FMF–Definition of colchicine resistance and literature review. **(B)** Previous treatments to IL-1 inhibitors. **(C)** Reasons for discontinuing steroids–Literature review. **(D)** Reasons for discontinuing anakinra. IL, interleukin; FMF, familial Mediterranean fever; MKD, mevalonate kinase deficiency; TRAPS, tumor necrosis factor receptor-associated periodic syndrome; LR, literature review.

Seven hundred thirty-one FMF patients (93.5%) were identified as colchicine-resistant. The frequency of febrile attacks, the maximum tolerated dose (1–3 mg/day), and patient adherence to treatment (**Figure 1A**) characterized colchicine resistance. The frequency of attacks was defined in n = 399 (54.6%) patients: ≥1/month (n = 67), ≥2/month (n = 24), ≥3/month (n = 228), ≥4/month (n = 19), ≥6/month (n = 7), and ≥12/month (n = 1). Colchicine toxicity was reported in 172 patients (21.8%), mainly diarrhea, neuromyositis, neutropenia, cardiomyopathy, myopathy, hepatic cytolysis, and asthenozoospermia.

### Other Previous Treatments

#### JIRcohort

Seven FMF patients (14%), 12 MKD patients (41.4%), and eight TRAPS patients (57.1%) were treated with immunosuppressive drugs and/or biological disease-modifying antirheumatic drugs (DMARDs) before IL-1 inhibitors, as detailed in **Figure 1B**. Reasons for discontinuation of previous treatments were ineffectiveness according to the treating physician (n = 16, 17.2%), a severe side effect (n = 2 for corticosteroids in an MKD and a TRAPS patient), or patient’s refusal (n = 1 MKD patient for corticosteroids). Reasons for discontinuation were not specified for nine patients (12.3%).

#### Literature Review

One hundred four FMF patients (13.2%), 117 MKD patients (44.2%), and 81 TRAPS patients (35.8%) were treated with immunosuppressive drugs and/or biotherapy before IL-1 inhibitors, as detailed in **Figure 1B**. Previous treatment was mainly stopped for therapeutic failure (n = 104 for FMF, n = 117 for MKD, and n = 81 for TRAPS). Nine patients (TRAPS, n = 8; MKD, n = 1) experienced a severe generalized urticarial rash, believed to be related to etanercept. Reasons for discontinuing steroid therapy are detailed in **Figure 1C**.

#### Reasons for Discontinuing Anakinra

Reasons for discontinuing anakinra are detailed in **Figure 1D** and were not specified for 7/24 patients (29.2%) in the JIRcohort. In the literature, the most common side effect was painful reactions at the injection site.

### Severe Clinical Complications

#### JIRcohort

**Figure 2:**
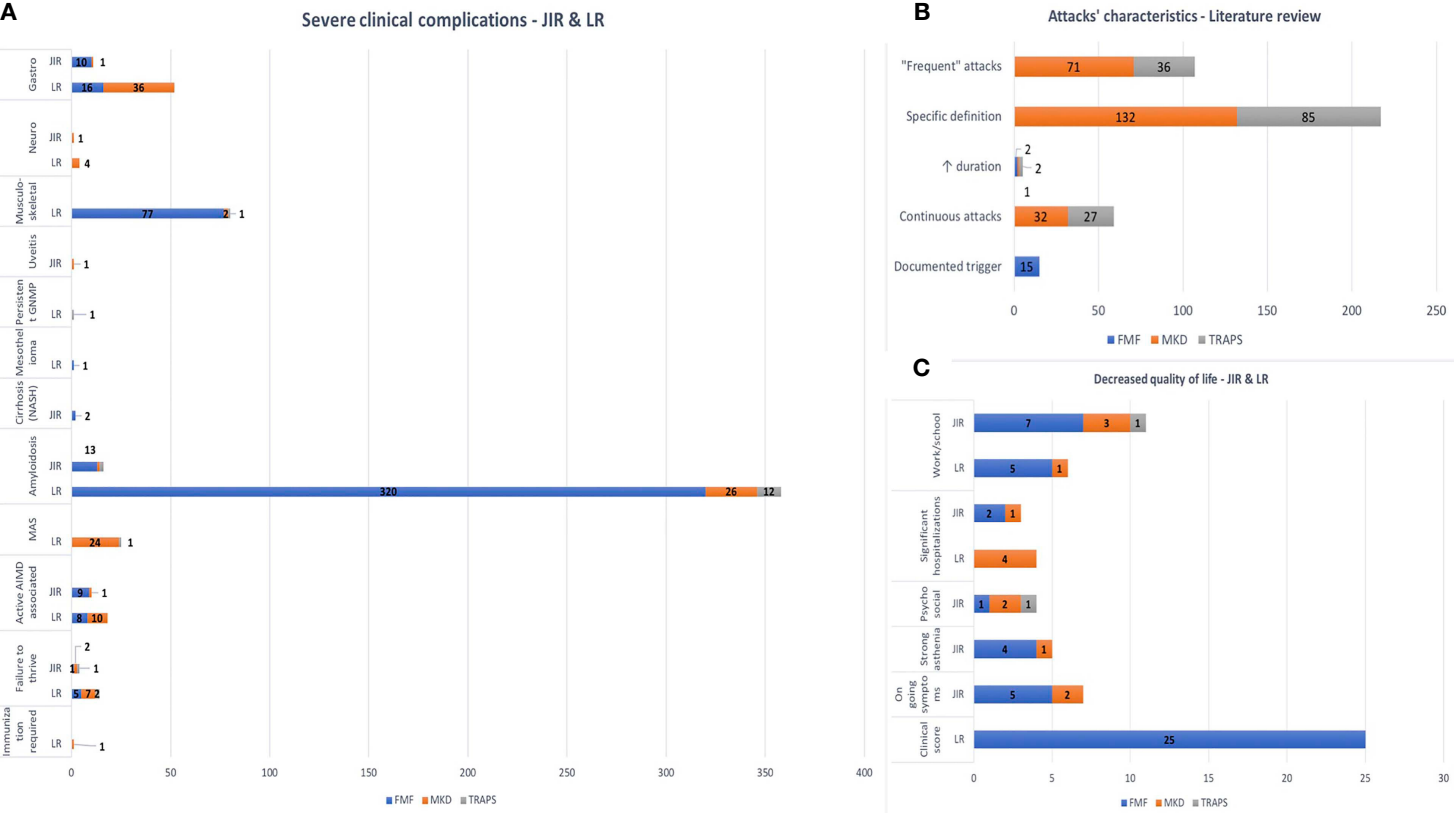
**(A)** Severe clinical complications. **(B)** Attacks’ characteristics–literature review. **(C)** Decreased quality of life. FMF, familial Mediterranean fever; MKD, mevalonate kinase deficiency; TRAPS, tumor necrosis factor receptor-associated periodic syndrome; LR, literature review; GNMP, membrano-proliferative glomerulonephritis; MAS, macrophage activation syndrome; AIMD, auto-immune disease.

Severe clinical complications or comorbidities prompted the introduction of IL-1 inhibitors in 29 FMF patients (58%), six MKD patients (20.7%), and three TRAPS patients (21.4%) in the JIRcohort (**Figure 2A**). Gastroenterological complications found in FMF were peritonitis (n = 7), severe chronic diarrhea (n = 2), ulcerative colitis (n = 1), and cirrhosis (n = 2). Cerebellar ataxia has been found in one MKD patient. Three FMF patients had active spondylitis, three had severe vasculitis (Henoch–Schönlein purpura or polyarteritis nodosa), one had Still’s disease, and two others had active inflammatory bowel disease.

#### Literature Review

Severe clinical complications or comorbidities prompted the introduction of IL-1 inhibitors in 305 FMF patients (38.6%), 62 MKD patients (23.4%), and 23 TRAPS patients (10.2%) (**Figure 2A**). Gastroenterological complications found in MKD were recurrent anal abscesses (n = 1), intestinal fistula and/or necrosis (n = 33), severe colitis (n = 4), and refractory ascites (n = 1). In FMF, severe colitis was found in 16 patients. Musculoskeletal symptoms were mainly reported in FMF patients, arthritis (n = 55) or protracted febrile myalgia (n = 22). Eighteen patients (1.5%) had an associated active inflammatory disease, leading to the introduction of IL-1 inhibitors: spondylitis (n = 1), Behçet syndrome (n = 6), and Henoch–Schönlein purpura (n = 1) for FMF patients and destructive polyarthritis (n = 10) for MKD patients. On-demand anakinra was used in one MKD patient to prevent a severe attack triggered by immunization.

### Biological Characteristics

#### JIRcohort

**Figure 3:**
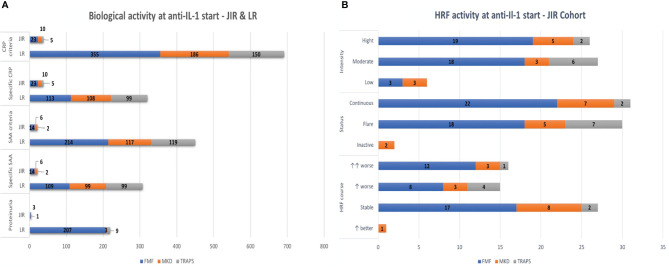
**(A)** Biological activity at IL-1 inhibitors’ start. **(B)** HRF activity at IL-1 inhibitors’ start. IL, interleukin; HRF, hereditary recurrent fever; FMF, familial Mediterranean fever; MKD, mevalonate kinase deficiency; TRAPS, tumor necrosis factor receptor-associated periodic syndrome; LR, literature review; CRP, C-reactive protein; SAA, serum amyloid A protein.

Persistent biological inflammation in-between attacks was reported in 46 patients (49.5%) including 27 FMF patients, 12 MKD patients, and seven TRAPS patients, mainly assessed by CRP levels >10 mg/L (**Figure 3A**).

#### Literature Review

The persistence of biological inflammation in-between attacks (n = 396, 30.9%) or its significant elevation during attacks (n = 363, 28.3%) led to the introduction of IL-1 inhibitors. Inflammation levels could be specific according to papers (CRP or SAA >10, 20, 25, or 30 mg/L) or any increased CRP/SAA level was sufficient (**Figure 3A**).

### Disease Activity

#### JIRcohort

Reported for only 19 patients (20.3% of the cohort), the VAS was >3/10 in seven FMF patients (14%), five MKD patients (17.2%), and one TRAPS patient (7.1%). Disease status, intensity, and time course were also assessed by the physician as described in **Figure 3B**.

#### Literature Review

The Physician Global Assessment (PGA) scale—used in most studies—was ≥2 for n = 190 patients (14.8%), and the VAS was reported for n = 118 patients (9.2%). The AIDAI score was exploited in 29 FMF and two TRAPS, and HRF activity was evaluated for 86 patients with a non-standardized score, which was different from one paper to another. These scores were consistently high but could not be compared with one another, as they were not standardized.

#### Inflammatory Flares’ Characteristics

In the literature, a documented trigger (n = 15, FMF), continuous attacks (n = 32, MKD and n = 27, TRAPS), prolonged (n = 1, MKD and n = 2, TRAPS) or frequent (n = 203, MKD and n = 121, TRAPS) attacks could justify the introduction of anakinra or canakinumab (**Figure 2B**).

### Quality of Life Assessment

#### JIRcohort

In 36 patients (38.7%; 19 FMF, 13 MKD, and four TRAPS), decreased quality of life contributed to the use of IL-1 inhibitors (**Figure 2C**). Two patients received on-demand anakinra to support school exams.

#### Literature Review

Decreased quality of life led to the start of IL-1 inhibitors in 100 patients (7.8%; 46 FMF, 46 MKD, and eight TRAPS) (**Figure 2C**). Different scores were used to evaluate the improvement of quality of life with IL-1 inhibitors: Short-Form 36 (SF36; n = 104 patients), Child Health Questionnaire-Parent Form 50 (CHQ-PF50; n = 32 patients), Health Assessment Questionnaire (HAQ; n = 13 patients), VAS (n = 30 patients), and social development (n = 71 patients).

## Discussion

To the best of our knowledge, this is the first paper describing extensively the criteria that lead in daily clinical practice to an indication of IL-1 blockers for adult and pediatric patients with FMF, MKD, and TRAPS. This work confirms that the reasons leading to the choice of treatment are considerably heterogeneous. However, four main categories seem to be consensual in both the JIRcohort and the literature: failure of previous treatment, a severe complication of the disease or associated comorbidity, persistent clinical and/or biological inflammation, and decrease in patients’ quality of life.

Persistent elevation of inflammatory markers and frequent attacks were the most common arguments for introducing IL-1 inhibitors. Inflammation was mainly judged on CRP levels, whose duration and severity depended on each prescriber or study. A clear definition of “frequent attacks” was rarely available, and when it did exist, the number of flares, duration, intensity, and time of onset were very heterogeneous. Recommendations for the management of MKD and TRAPS are also imprecise, as IL-1 inhibitors are required for “frequent attacks and/or subclinical inflammation between attacks” (123–125).

For FMF, IL-1 blockers are indicated only for colchicine resistance or intolerance; however, there is currently no consensus in the literature for a clear definition of crFMF. According to the Franco-Israeli consortium, crFMF is defined as the occurrence of six or more typical attacks in 1 year or three in 4–6 months associated with an increase in inflammatory markers between attacks (126); according to European Alliance of Associations for Rheumatology (EULAR) definition, as at least one flare per month in the last 6 months with full compliance to treatment (127) and as more than six attacks per year or more than four attacks in the last 6 months with persistent biological inflammation (128). Recently, Ozen et al. (129) agreed that crFMF included recurrent attacks (one or more attacks per month over 3 months) or persistent laboratory inflammation in-between attacks. Eighty-two percent of patients receiving IL1 inhibitors were considered crFMF in our study, similar to results (89%) of Kacar et al. (130). However, many factors could contribute to insufficient response to colchicine: lack of compliance, the occurrence of side effects, drug interactions, genetic factors, and environmental factors (infections, stress, and diet) (131). All studies and definitions agree that a maximum tolerated dosing of colchicine (132) and the assessment of compliance are the prerequisites before discussing crFMF (133), but finding a standardized way is challenging (134). Missing tablet count, Morisky score (135), and colchicine dosage in the hair were rarely reported in our study. Hair colchicine testing was recently proposed (136) to assess objectively and non-invasively adherence from 2 to 6 months before the sampling. Toxicity of colchicine is rare in our study, as approved by experts in the literature (129).

Our study showed that only a proportion of patients appear to require IL-1 inhibition. Unfortunately, it was not feasible to estimate the exact ratio of patients requiring these treatments because we did not have the opportunity to verify that all patients from participating centers were included in the cohort. However, IL-1 inhibitors do not appear to be offered as first-line therapy to all patients in daily practice. One reason could be that at the time of the persistent HRF activity, IL-1 inhibitors were not always available in all centers. Steroids and TNF-α blockers were mainly used before IL-1 inhibitors in our study. Steroids can be used shortly for flares’ treatment but may only have partial efficacy in MKD (10, 100) and a declining efficacy in TRAPS, requiring a higher dose for an equivalent response with many side effects (125). Efficacy of etanercept in TRAPS patients appears transient and decreases over time (137, 138), confirmed in our study with the reappearance of inflammatory flares. A better clinical and biological response also has been described by Ozen et al. (96) with an IL-1 rather than a TNFα inhibitor in TRAPS patients on first-line therapy, which also represent by far the most frequently employed biologics in the Auto-Inflammatory Diseases Alliance (AIDA) network (139). IL-1 inhibitors could be recommended as first-line therapy and for cortisone sparing in severe phenotypes (96).

A severe phenotype of HRF justified IL-1 inhibitors in about 30% of patients in the JIRcohort and the literature. Active inflammatory comorbidities found in our study have been known to be associated with FMF, vasculitis [Behçet’s disease (140), Henoch–Schönlein purpura, periarteritis nodosa (141)], spondylitis (142), and suppurative hidradenitis (143), whereas inflammatory bowel diseases (144) and multiple sclerosis (145) are still controversial. As recommended (127), intensified treatment with biological DMARDs was given in 26% of FMF patients with secondary amyloidosis in the JIRcohort. Similar results (20%) were found in the study by Corsia et al. (128). Such a therapeutic strategy has proven its effectiveness, as shown by a recent study, in which treatment with canakinumab was significantly associated with decreased proteinuria in secondary amyloidosis patients (111). In TRAPS patients complicated with amyloidosis, especially in the case of structural mutation involving cysteine (124), IL-1 inhibitors also appeared to be the most effective treatment (41, 146–148). The majority of patients in our cohort as well as those in the literature requiring IL-1 inhibition had a confirmatory genotype according to the International Study Group for Systemic Autoinflammatory Diseases (INSAID) rules. The need for this type of treatment in patients with a non-confirmatory genotype [heterozygous FMF or MKD without mevalonic aciduria, presence of a variant of uncertain significance (VOUS) in TRAPS] seems much more uncommon. The few patients classified in our cohort as having “no mutation” concern patients (i) for whom the diagnosis was made before the generalization of genetic testing and who were never tested afterward or (ii) for whom genetics was missing data.

Several (sometimes non-standardized) scores were recorded to measure disease activity and lead to IL-1 inhibitor treatment. VAS, the most frequently used score in the JIRcohort, has the disadvantage of trying to assign a single value to a complex and subjective perception of the disease, causing disparate therapeutic decisions depending on the prescriber. The FMF50, which defines non-responders to colchicine (149), and adult (150, 151) or pediatric (152) severity scores such as the International Severity Score for FMF (ISSF) are not very sensitive (153). With a sensitivity and specificity of more than 80%, the AIDAI score differentiates patients with active disease (score >9) from those with inactive disease (score <9) (154). Despite its validation and possible use in daily practice, it seems difficult to have it completed daily by patients. Furthermore, the AIDAI score has not yet been validated in a treat-to-target approach: the maximal cutoff value and the specificity of the score for the inflammatory manifestations are still unknown.

There is currently no score evaluating disease activity from both physician’s and patient’s perspective, taking into account the quality of life, which was a strong argument in our study for introducing IL-1 inhibitors. Several authors confirmed a poorer quality of life in children and adults with FMF (155–158) and a higher incidence of anxiety and depression (159–161) that may lead to more frequent attacks with higher rates of CRP and SAA (159, 161, 162). Van der Hilst et al. (10) also described lower autonomy and social development in MKD patients. Estimating quality of life in HRF is complex, involved disease activity and severity, and would require a standardized assessment to justify therapeutic escalation and improve management.

Even if canakinumab is today the only IL-1 inhibitor to have MA in Europe for treatment of MKD and TRAPS, nearly two-thirds of patients in our cohort were treated with anakinra as a first-line. There is so far no data comparing the two drugs that both seem to be effective in these HRFs (9–12, 32, 46, 54, 55, 60, 100, 104, 105, 133, 163, 164), but their costs differ. Anakinra has a shorter half-life, limiting drug overdosing in patients with renal impairment (94, 113, 165), and the French–Israeli expert committee recommends its use for crFMF patients before considering a long-half-life IL-1 inhibitor (126). Canakinumab is an alternative in case of poor tolerance of daily anakinra injections. The efficacy of anakinra prescribed on demand has been described in cases of known triggers [stress of an examination or vaccinations (56)] or rare inflammatory flares (78, 84, 103). On-demand prescribing may limit local reactions to injections, decrease the use of steroids and the risk of infection, but there are currently no studies comparing anakinra on-demand *vs.* continuous use. However, experts recommend continuous treatment for persistent inflammatory syndrome between attacks or the use of anakinra on demand more than once a month (123, 124). Considering its benefits and efficacy, anakinra may also soon be granted MA for MKD and TRAPS.

The major flaw of our study is its retrospective design. Since the collection of data in the JIRcohort began in April 2016, the clinical, biological, and quality of life characteristics of patients treated with IL-1 inhibitors before this date were not captured. The heterogeneity of experience in managing HRF patients among different centers and the lack of follow-up tools in the JIRcohort are also part of the limitations of our study. The strengths of our study are the large sample of both children and adults and its multicentric dimension, including countries with different market authorizations.

## Conclusion

Our study has shown that the indications in real life for the use of IL-1 inhibitors in crFMF, MKD, and TRAPS are still not standardized and poorly defined. The choice for treatment with IL-1 inhibitors is based on the habits of expert clinicians that may vary from one center to another. Furthermore, the availability of the drugs may vary from country to country. Several prerequisites will be necessary to harmonize clinical care: (i) a formalization of the different criteria that clinicians use to set the indication for IL-1 inhibition in HRFs, (ii) validated composite activity scores that will eventually help to define treat-to-target treatment plans, (iii) a comparison of the different treat-to-target strategies on the actual long-term outcome of the patients, and (iv) a medico-economic analysis of the different possible treatment plans. We recently launched a European-wide survey to collect treatment plans and outcome measures applied to patients with HRF. We will hence provide a snapshot of current care and clinical practice strategies in Europe. In the long term, comparing the different strategies used will guarantee the patients with autoinflammatory diseases to be offered the optimal treatment available in their country.

## Data Availability Statement

The original contributions presented in the study are included in the article. Further inquiries can be directed to the corresponding author.

## Author Contributions

All authors participated in the preparation of the manuscript. All authors contributed to the article and approved the submitted version.

## Conflict of Interest

The authors declare that the research was conducted in the absence of any commercial or financial relationships that could be construed as a potential conflict of interest.

## Publisher’s Note

All claims expressed in this article are solely those of the authors and do not necessarily represent those of their affiliated organizations, or those of the publisher, the editors and the reviewers. Any product that may be evaluated in this article, or claim that may be made by its manufacturer, is not guaranteed or endorsed by the publisher.
